# Effect of Rapid Hollow Cathode Plasma Nitriding Treatment on Corrosion Resistance and Friction Performance of AISI 304 Stainless Steel

**DOI:** 10.3390/ma16247616

**Published:** 2023-12-12

**Authors:** Jinpeng Lu, Haichun Dou, Zelong Zhou, Haihong Li, Zhengwei Wang, Mingquan Jiang, Fengjiao Li, Yue Gao, Chenyu Song, Dazhen Fang, Yongyong He, Yang Li

**Affiliations:** 1 College of Nuclear Equipment and Nuclear Engineering, Yantai University, Yantai 264005, China; ytuljp@163.com (J.L.); dhc7884@163.com (H.D.); zhouzelong20000313@163.com (Z.Z.); hhli@ytu.edu.cn (H.L.); jmqcccccc@163.com (M.J.); lifengjiao@163.com (F.L.); ggyy44777@163.com (Y.G.); 13153861991@163.com (C.S.); 2 State Key Laboratory of Tribology in Advanced Equipment, Tsinghua University, Beijing 100084, China; wangzwaixuexi@163.com (Z.W.); fdz21@mails.tsinghua.edu.cn (D.F.)

**Keywords:** austenitic stainless steel, corrosion, hollow cathode, plasma nitriding, friction properties

## Abstract

Low-temperature plasma nitriding of austenitic stainless steel can ensure that its corrosion resistance does not deteriorate, improving surface hardness and wear performance. Nevertheless, it requires a longer processing time. The hollow cathode discharge effect helps increase the plasma density quickly while radiatively heating the workpiece. This work is based on the hollow cathode discharge effect to perform a rapid nitriding strengthening treatment on AISI 304 stainless steels. The experiments were conducted at three different temperatures (450, 475, and 500 °C) for 1 h in an ammonia atmosphere. The samples were characterized using various techniques, including SEM, AFM, XPS, XRD, and micro-hardness measurement. Potentiodynamic polarization and electrochemical impedance spectroscopy methods were employed to assess the electrochemical behavior of the different samples in a 3.5% NaCl solution. The finding suggests that rapid hollow cathode plasma nitriding can enhance the hardness, wear resistance, and corrosion properties of AISI 304 stainless steel.

## 1. Introduction

Austenitic stainless steel finds broad applicability in various industries, such as aerospace, chemical, medical, and marine engineering, primarily owing to its exceptional resistance to corrosion [1,2,3]. However, the mechanical and frictional characteristics of austenitic stainless steel present inherent limitations that may result in friction-induced damage to the workpiece, particularly in corrosive environments [4,5,6]. Plasma nitriding has the advantages of high efficiency, low cost, and less pollution [7,8,9,10,11]. It is a standard surface-strengthening treatment method for austenitic stainless steel [12,13,14,15,16]. Many N-containing gases are ionized, nitrogen ions and nitrogen active substances collide and deposit on the surface, and the N element diffuses into the interior and forms nitrides. This nitride is a solid solution phase with a high N element, commonly known as the expanded austenite S-phase [17,18,19].

The presence of the S-phase has been found to enhance the surface hardness, corrosion resistance, and wear resistance of austenitic stainless steels [20,21,22,23]. In traditional plasma nitriding, the edge effect will appear on the surface, resulting in significant differences in the structure and properties [24]. There are currently various types of nitriding used to strengthen AISI 304 austenitic stainless steel, mainly including triode-plasma nitriding [25], radio frequency (rf) plasma nitriding [26], nitrogen plasma immersion ion implantation [27], low-pressure hollow cathode plasma source [28], cathodic cage plasma nitriding [29,30,31], and hollow cathode-assisted plasma nitriding [32,33]. It is important to note that these techniques are applied at temperatures below 450 °C. These techniques above a temperature threshold of 475 °C may lead to chromium nitride (CrN) precipitation formation. This occurrence induces a condition known as “poor chromium,” which diminishes corrosion resistance [34,35]. Unfortunately, the diffusion coefficient of nitrogen within the matrix is temperature dependent. Low temperature makes it difficult to provide practical help for the diffusion of nitrogen in the matrix, which leads to nitrogen having difficulty penetrating the matrix [36].

The hollow cathode plasma nitriding technology exploits the distinctive geometric properties of a hollow cathode to create multiple overlaps of the negative glow region in the glow discharge. It results in an intensified ionization of gas molecules, giving rise to an “avalanche” effect. Consequently, the ionization rate and plasma density experience a significant increase, leading to a bombardment of the cathode by numerous ions and neutral particles, hence causing a rapid elevation in temperature [37]. By altering the geometric configuration of the hollow cathode, it becomes possible to achieve a higher yet controllable temperature within a short period. Zhang [38] used COMSOL v5.6 software to simulate the plasma physical field formed by the hollow cathode structure. The results show that the hollow cathode mechanism can effectively increase the plasma density and make the sample in a high plasma density environment, which is beneficial to the nitriding process. Leyland et al. [39] believed that the cathode potential required for surface activation is much lower than that used for heating. When heated rapidly in the cathode state, the austenitic stainless steel can effectively avoid the ‘poor chromium’ phenomenon and quickly obtain a better strengthening effect. K. Nikolov et al. [40] carried out a short-time plasma nitriding of austenitic stainless steel using a hollow cathode structure. The results show that the hollow cathode can also perform controllable nitriding treatment on austenitic stainless steel and obtain a modified layer with better performance.

In this study, we aimed to enhance the surface properties of austenitic stainless steel with a newly designed hollow cathode structure device. AISI 304 stainless steel was subjected to rapid hollow cathode plasma nitriding to attain superior mechanical and friction properties without compromising the inherent corrosion resistance.

## 2. Materials and Methods

The utilized material is a rolled AISI 304 austenitic stainless steel plate with a diameter of 25 mm and a thickness of 8 mm. The chemical composition is shown in Table 1. The surface of the AISI 304 substrate was polished with up to 2000 silicon carbide sandpaper and 3.5 μm diamond polishing solution, and the surface roughness was less than 0.08 μm. To prevent the impurities in the grinding and polishing process from affecting the subsequent treatment, acetone and anhydrous ethanol were used to clean the matrix ultrasonically and then dried.

Hollow cathode-assisted rapid plasma nitriding experiments were conducted using an LDMC-20 plasma nitriding device [41]. The sample was placed on the working table of the nitriding furnace, and the pressure was reduced to below 30 Pa. Argon gas was then introduced to sputter the sample surface for 15 min, serving as a secondary cleaning step. The voltage was set to 750 V, with a duty cycle of 73%. NH_3_ was introduced into the furnace at a flow rate of 300 sccm until the pressure reached 300 Pa for heat preservation. The nitrided temperature was consecutively manipulated at 450 °C (LTPN), 475 °C (MTPN), and 500 °C (HTPN).

The phase composition was conducted using a Bruker D8 ADVANCE X-ray diffractometer (XRD). The instrument utilizes Cu-Kα radiation as the X-ray source. The scanning range was set from 20° to 90°with a 4°/min scanning rate. The XRD instrument was operated at an accelerating voltage of 40 kV and a current of 40 mA. These parameters ensured the generation of accurate and reliable diffraction patterns from the analyzed samples. The surface and cross-sectional morphology were observed using an optical microscope (OM, Axio Observer 3 materials, ZEISS, Oberkochen, Germany), and the thickness of the S-phase was measured. The wear morphology and surface roughness of the sample before and after wear were analyzed by a three-position white light interference topography instrument (NeXView, ZYGO, Middlefield, CT, USA). A NanoScope III atomic force microscope (AFM, MFP-3D-SA, Asylum, Oxford, MS, USA) was used to study the detailed surface morphology in cyclic strain.

The surface hardness was evaluated using a Duramin-A300Vickers hardness tester model. Multiple measurements were taken, and the average value was recorded. The test load applied was 100 g-force (gf), and the duration of the indentation was kept constant at 15 s. X-ray photoelectron spectroscopy (XPS, PHI Quantera II, Ulvac-Phi, Chigasaki, Japan) was used to analyze the surface phase of the sample after corrosion. Al-Kα, with an operating power of 26.3 W, was used as an X-ray source. The incident angle was 45°, and the excitation energy was 280.00 eV. To ascertain the nano-hardness and elastic modulus of the various samples, a nanoindentation test was conducted utilizing a nanohardness tester (NHT3, Anton Paar, Graz, Austria). Reciprocating friction experiments were conducted using a model UMT-5 friction and wear tester (Bruker, Billerica, MA, USA). It was conducted for 20 min, and the frequency was set at 2 Hz. A load of 5 N was applied, and the friction pair was a 4 mm diameter Al_2_O_3_ ball.

An electrochemical workstation (VersaSTAT 3F, Ametek, Shanghai, China) was used to conduct a series of electrochemical tests, including an open circuit potential test, potentiodynamic polarization, and electrochemical impedance spectroscopy (EIS) test, to evaluate the corrosion behavior of the sample in 3.5% NaCl solution environment. The traditional three-electrode system was used for the corrosion test. The working electrode consisted of a saturated calomel electrode (SCE) immersed in a saturated potassium chloride solution that served as the reference electrode. A platinum mesh electrode was employed as the counter electrode. Before all experiments, the samples were immersed in 3.5% NaCl solution for 1 h, and electrochemical stability was achieved at open circuit potential (OCP). Potentiodynamic polarization tests were performed from −1.0 V_SCE_ to 1.2 V_SCE_. Electrochemical impedance spectroscopy (EIS) measurements were performed under OCP with a frequency of 10^5^ to 10^−2^ Hz.

## 3. Results and Discussion

**Figure 1:**
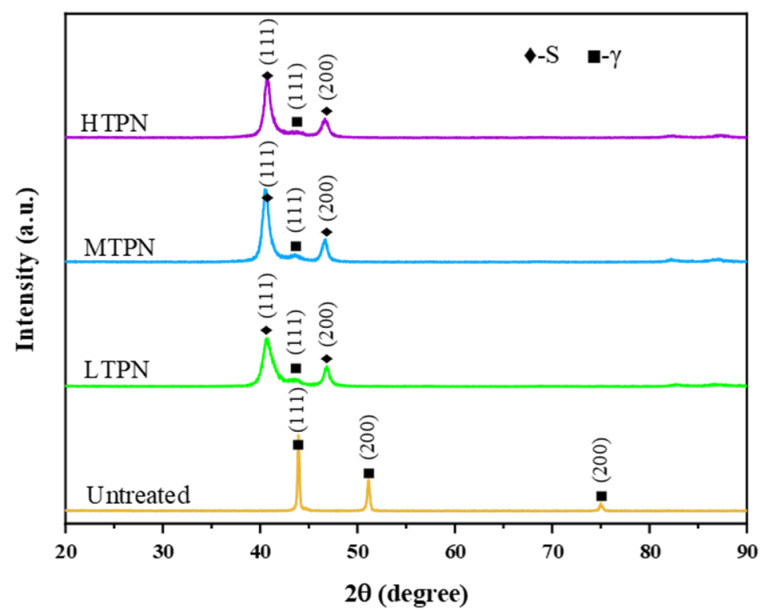
X-ray diffraction analysis for the untreated and treated samples.

Figure 1 shows the X-ray diffraction analysis for the untreated and treated samples. It was found that the untreated sample exhibited a typical face-centered cubic structure (fcc), and the γ phase was located at 44°, 51°, and 75°, respectively [42]. The XRD patterns of the nitrided samples show a substantial similarity. Only a very weak γ phase peak was found at 44°, and firm S-phase peaks were found at 41° and 47° for the nitrided samples. The formation of the S-phase is attributed to the expansion of the austenitic lattice resulting from the dissolution of nitrogen [43,44]. However, due to the short nitriding time, X-rays can penetrate the thinner S-phase layer and can still be observed in the γ phase. The increase in temperature leads to more nitrogen infiltration into the matrix, leading to lattice expansion. Consequently, the peak corresponding to the S-phase tends to shift towards lower diffraction angles.

**Figure 2:**
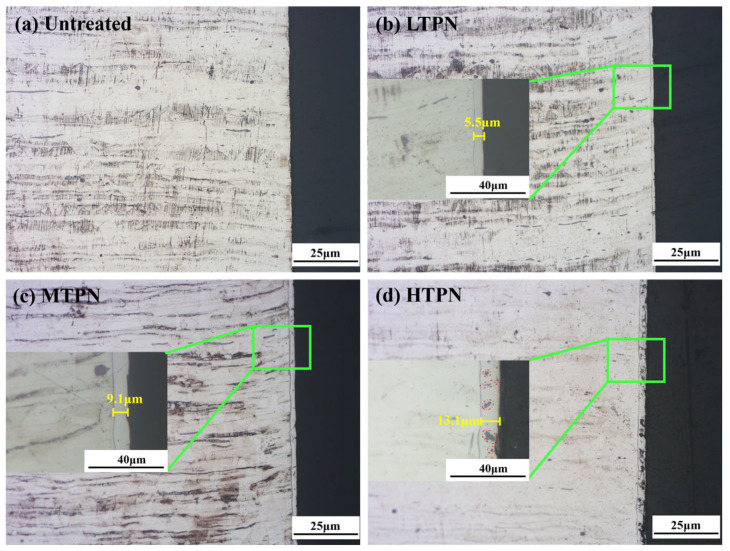
Cross-sectional OM micrographs of the untreated and the treated sample: (**a**) untreated, (**b**) LTPN, (**c**) MTPN and (**d**) HTPN.

Figure 2 shows the cross-sectional micrographs of the various samples, followed by etching with a chemical solution containing 10 g CuSO_4_·5H_2_O, 2 mL H_2_SO_4_, 40 mL HCl, and 40 mL H_2_O. It revealed a white layer on the nitrided surface, expressly referred to as the S-phase layer [21]. As increase in the nitriding temperature corresponded to an augmentation in the thickness of the S-phase layer, extending from 5.5 to 13.1 μm. Novel plasma nitriding techniques incorporating rare earth catalysis, plasma implantation, and laser nitriding have proven effective in enhancing the nitrided layer thickness and surface performance of austenitic stainless steel. Nevertheless, these methodologies still necessitate high temperatures or prolonged processing times, leading to inefficiencies. Current research on conventional plasma nitriding indicates that the formation of about 5–6 um thickness S-phase commonly requires 4 h or longer [33]. Conversely, utilizing rapid hollow cathode plasma nitriding technology, a nitrided layer thickness of approximately 5.5μm can be achieved after just 1 h of processing. This notable difference in processing time clearly illustrates the distinct advantages of rapid hollow cathode plasma nitriding technology.

In the metallographic images of the HTPN sample (Figure 2d), distinct black spot-like substances, identified as precipitated CrN, were observable within the S-phase layer [45]. However, the XRD pattern of the HTPN sample displayed no evidence of the CrN phase (Figure 1). It can be explained by the fact that the precipitation of CrN must reach a certain volume fraction to generate detectable XRD reflections [33].

**Figure 3:**
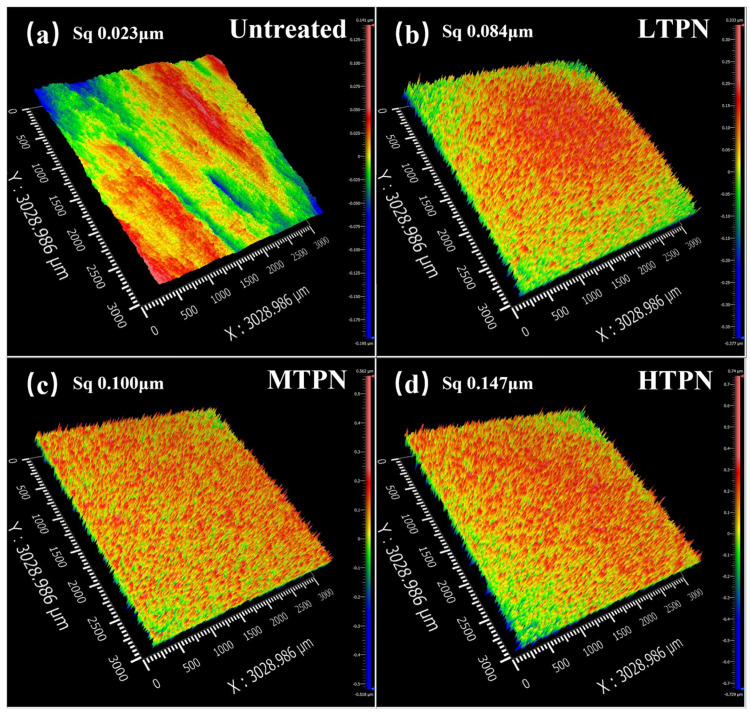
The three-dimensional white light interferogram of the surface of the untreated and the treated sample: (**a**) untreated, (**b**) LTPN, (**c**) MTPN and (**d**) HTPN.

Figure 3 presents a three-dimensional white light interferogram illustrating the surface characteristics. It was determined that the untreated sample (Figure 3a) exhibited the lowest surface roughness value of 0.023 μm due to the mechanical grinding and polishing. The LTPN, MTPN, and HTPN samples exhibited surface roughness values of 0.084, 0.100, and 0.147 μm, respectively. Moreover, it was observed that the surface roughness of the samples escalated in tandem with the rise in nitriding temperature. It can be explained by the heightened temperature experienced during the nitriding process, which intensifies particle motion and enhances the energy imparted to the surface through bombardment. Additionally, incorporating nitrogen elements into the solid solution results in lattice expansion, further contributing to the increased surface roughness [46].

**Figure 4:**
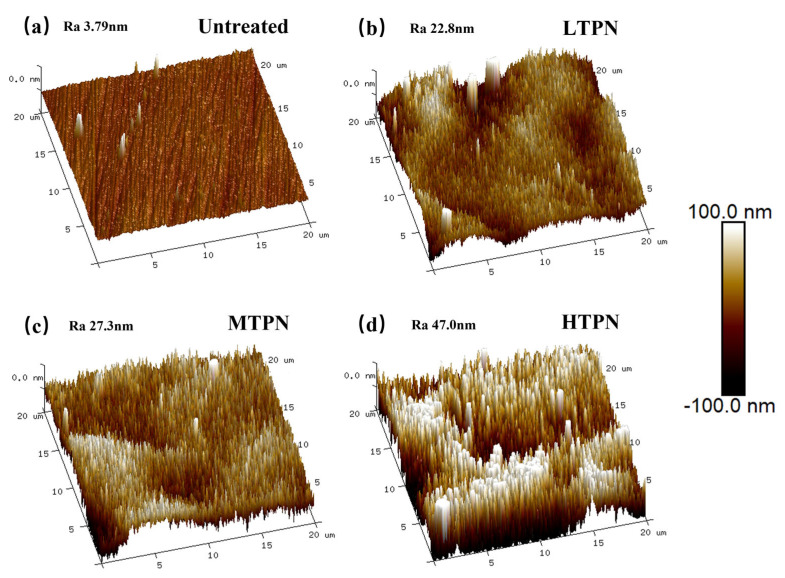
The AFM images of the untreated and the treated samples: (**a**) untreated, (**b**) LTPN, (**c**) MTPN and (**d**) HTPN.

The study employed AFM to investigate the surface characteristics of the nitrided samples, as depicted in Figure 4. As depicted in Figure 4a, the surface morphology of the untreated sample exhibits a predominantly smooth texture with minimal protruding spikes. Conversely, the nitrided samples display a considerably higher density of spikes. M. Sharif [47] postulated that the deposition of the sputtering material for the auxiliary cathode device resulted in the appearance of these structures. The obtained analysis reveals that the untreated sample manifests a surface roughness value of merely 3.79 nm.

As the temperature increases, the surface bombardment intensifies, and the diffusion ability of the N element in the solid solution is enhanced, which further causes the lattice to expand and increases the surface roughness [48]. This finding is consistent with the results obtained from three-dimensional white light interferometry analysis (Figure 3). It can be observed that there are some depressions and reliefs on the nitrided surface in Figure 4. It is mainly due to the solid solution of a large amount of nitrogen element, which causes local plastic deformation and produces internal stress. The higher internal stress will form slip steps and reliefs at the grain boundary [49].

**Figure 5:**
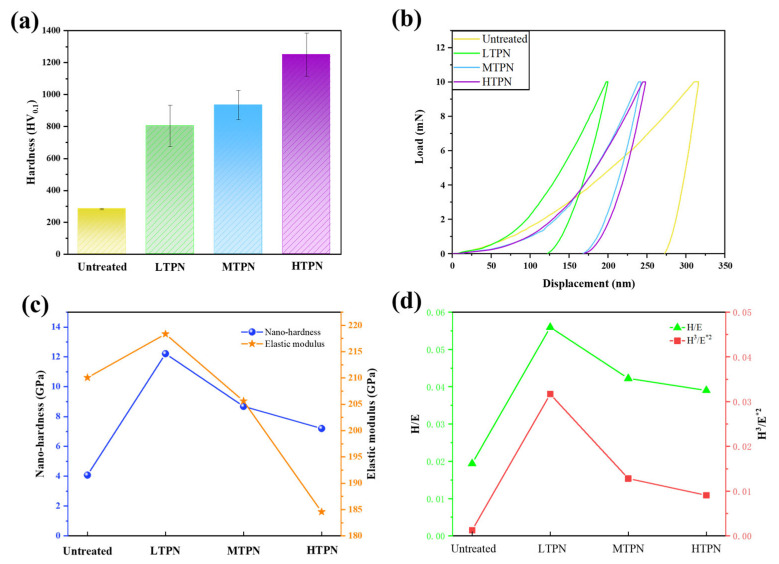
(**a**) Surface microhardness, (**b**) load-displacement curve, (**c**) nano-hardness and elastic modulus, and (**d**) H/E and H^3^/E^⁎2^ ratios derived from the nanohardness and elastic moduli.

Figure 5a shows the surface microhardness of different samples. A comparison indicated that the untreated sample exhibits a significantly low surface hardness of merely 283 HV_0.1_. In contrast, the surface hardness of the sample significantly increased after nitriding treatment. To be precise, the surface hardness values for the LTPN and MTPN samples measured 805 HV_0.1_ and 935 HV_0.1_, respectively. The HTPN sample attained the highest surface hardness level of 1250 HV_0.1_, primarily attributed to complex nitrides within the S-phase [20]. The enhancement in the hardness is ascribed to the gap solid solution hardness mechanism and the consequential rise in residual stress resulting from the formation of the expanded austenite phase [50]. Concerning the micro-level mechanical properties, the ranking order is as follows: Untreated < LTPN < MTPN < HTPN.

Nanoindentation tests were conducted further to evaluate the mechanical properties of the nitrided sample surfaces. Figure 5b illustrates the load-displacement curve obtained from the nanoindentation tests for the different samples. Based on the obtained data, the stiffness ranking of the samples could be ascertained as follows: Untreated < HTPN < MTPN < LTPN. Figure 5c compares the nano-hardness (H) and elastic modulus (E) values of the different samples. The LTPN sample demonstrated superior nano-hardness and elastic modulus values, measuring 12.2 GPa and 218.3 GPa, respectively.

Conversely, the untreated sample exhibited the lowest nano-hardness and elastic modulus values, measuring 4.1 GPa and 210.0 GPa, respectively. This disparity can be attributed to the infiltration of nitrogen elements, leading to an increase in residual stress [44,51]. Figure 5d exhibits the H/E and H^3^/E*^2^ values of the different samples. These values can indicate resistance to elastic strain failure and plastic deformation. Larger values reflect a more substantial capacity of the sample surface to endure elastic strain failure and plastic deformation [33]. Similarly, the order is as follows for the nano-level mechanical properties: Untreated < HTPN < MTPN < LTPN.

**Figure 6:**
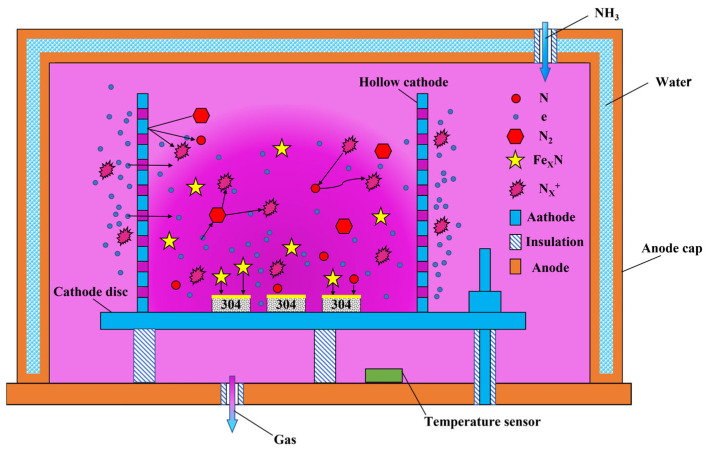
Schematic of the principle processes of nitrogen mass transfer in the hollow cathode plasma nitriding treatment.

The experimental setup operated in a low-pressure working atmosphere filled with NH_3_, as depicted in Figure 6. As NH_3_ gas was ionized, the particles started to move and collide. The unique structure of the hollow cathode promotes a back-and-forth oscillation of electrons between the circular hole and the cathode barrel wall, thereby increasing the likelihood of particle collision, enhancing ionization efficiency, and intensifying discharge. Due to the large surface area of the hollow cathode barrel, a substantial proportion of positive ions no longer directly bombard the surface. Instead, many iron atoms can be sputtered and combined with the active nitrogen in the atmosphere. The resulting active iron nitride adsorbs onto the substrate surface. Active nitrogen substance diffuses into the substrate and combines with the existing substances to form a nitrided layer. This process is called the “sputtering-deposition-adsorption-diffusion” mechanism for nitrogen mass transfer.

**Figure 7:**
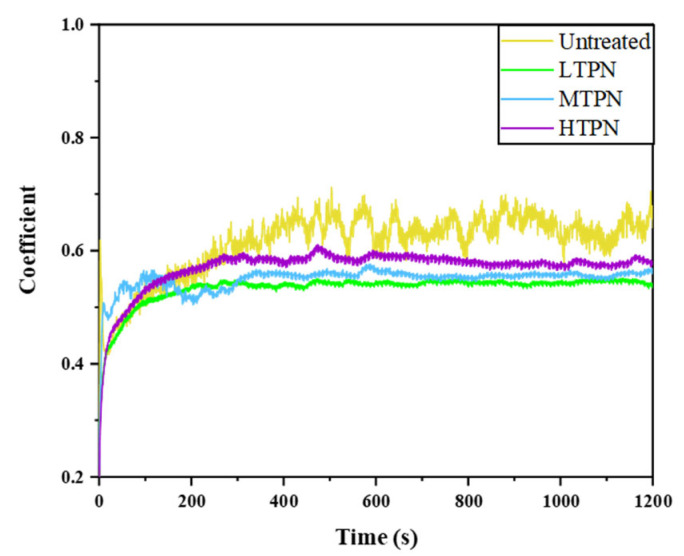
Coefficient of friction versus time for the untreated and treated samples.

Figure 7 presents the friction coefficient (COF)of the different samples. The untreated 304 steels exhibited an average friction coefficient of approximately 0.61. Notably, the COF curve showed significant fluctuations beyond 300 s. Compared with the Al_2_O_3_ ball, the hardness of the untreated substrate surface was relatively lower. Plastic deformation occurred on the substrate during relative motion and more pronounced fluctuations. The insufficient surface hardness of the substrate contributed to the detachment of surface material by the higher hardness Al_2_O_3_ ball, leading to the adherence of this material to the surface of the Al_2_O_3_ ball and further deterioration of friction performance. The observed plastic deformation on the untreated surface can result from their relatively low elastic modulus.

The nitrided samples exhibited a low friction coefficient, and the overall curve showed less pronounced fluctuations. The surface hardness of the nitrided samples exceeded the hardness of the Al_2_O_3_ ball, causing a change in the friction mechanism. The average friction coefficient of the LTPN, MTPN, and HTPN samples were 0.52, 0.55, and 0.58, respectively. The LTPN sample displayed a lower friction coefficient and more stable fluctuation. The anti-wear performance of the samples can be ranked as follows: Untreated < HTPN < MTPN < LTPN.

Figure 5 demonstrates that the nitrided sample significantly improves microhardness, nano-hardness, and H^3^/E^⁎2^ compared to the untreated sample. These enhanced mechanical properties contribute to the ability to withstand deformation and resist the cutting effects of the Al_2_O_3_ ball.

**Figure 8:**
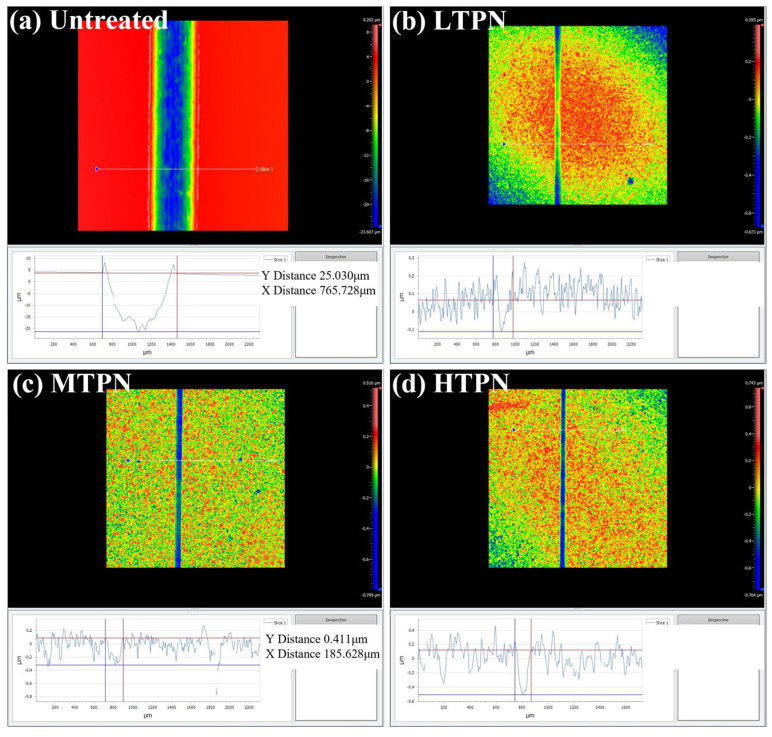
Three-dimensional white light interferograms of wear scars on the different samples: (**a**) untreated, (**b**) LTPN, (**c**) MTPN and (**d**) HTPN.

**Figure 9:**
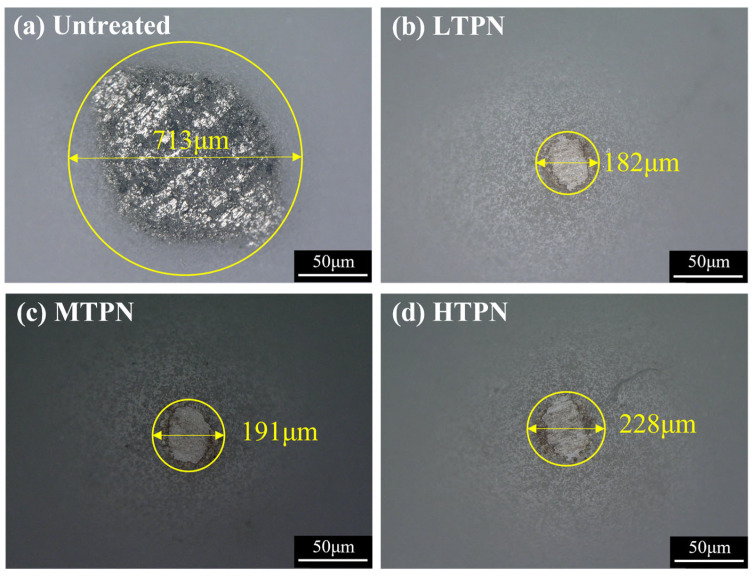
OM micrographs of wear tracks on Al_2_O_3_ ball corresponding to the untreated and nitrided samples after friction: (**a**) untreated, (**b**) LTPN, (**c**) MTPN and (**d**) HTPN.

In order to study the mechanism of friction damage, three-dimensional white light interferograms of wear scars on the different samples (Figure 8) and OM micrographs of wear tracks on Al_2_O_3_ balls corresponding to the different samples after friction were obtained (Figure 9). The wear scar of the untreated sample (Figure 8a) exhibited the broadest width and most profound depth, measuring 25.03 and 765.73 μm, respectively. The nitrided sample’s wear scar width and depth were significantly reduced. The wear scars observed on the surface of the untreated sample (Figure 8a) exhibited distinct U-shaped grooves, with evidence of plastic deformation at the edges of the wear scars. Additionally, Figure 9a reveals that the surface of the Al_2_O_3_ spheres remains un-worn but exhibits significant adhesion. This phenomenon arises from removing the delaminated material from the untreated surface by the reciprocating Al_2_O_3_ spheres, which subsequently adhere to the surface of the spheres. The wear characteristics of the untreated sample mainly manifested as plastic deformation and adhesive wear. In contrast, the wear scars on the nitrided samples exhibited shallow and deep grooves, with a lack of plastic deformation at the edges of the wear scars, indicating a change in the wear mechanism. The presence of nitrides leads to an increase in surface hardness, causing wear on the surface of the Al_2_O_3_ ball. This outcome arises from the highly superior hardness of the nitrided layer relative to that of the Al_2_O_3_ ball, as confirmed by the increase in surface hardness of the nitrided sample (Figure 5a). Multiple furrows were also evident at the wear site of the Al_2_O_3_ ball. The nitrided samples exhibited a typical abrasive wear mechanism. This phenomenon is due to the diffusion of nitrogen atoms on the substrate surface during the nitriding process, forming a nitrided layer [52]. P. Ruiz-Trabolsi [53,54,55] indicated that the untreated sample displays a soft surface that undergoes plastic deformation upon relative motion due to surface squeezing. Moreover, the application of shear force causes the surface to be peeled off and adhere to the Al_2_O_3_ ball, resulting in adhesive wear. In contrast, the nitriding sample exhibited a substantial amount of nitride on its surface which enhances surface hardness and wear resistance. During friction, the protruding peaks on the micro-surface of the nitriding sample were abraded and functioned as abrasives between the friction pairs, leading to abrasive wear. Furthermore, the diffusion of nitrogen on the surface of the nitrided sample induced solid solution strengthening, effectively preventing plastic deformation.

The wear volume and wear rate of the sample were calculated in Table 2. The untreated sample exhibited poor wear resistance, as evidenced by a 2.30 × 10^2^ mm^3^ wear volume and a 1.92 × 10^−3^ mm^3^/Nm wear rate. Following nitriding, a substantial improvement in wear resistance was observed. Compared to the untreated sample, the nitrided sample displayed a wear rate reduced by three orders of magnitude, accompanied by a commensurate decrease in wear volume. These findings indicate that the S-phase can enhance the wear resistance of AISI 304. The LTPN sample exhibited the smallest wear volume and rate among the nitrided samples, indicating superior wear resistance.

**Figure 10:**
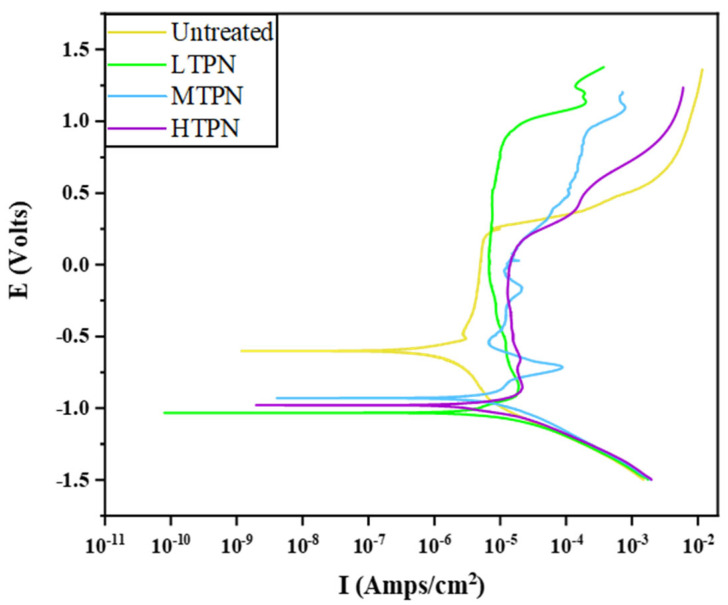
Polarization curves of the untreated and treated samples in 3.5  wt% NaCl solution.

Figure 10 illustrates the polarization curves obtained for the different samples. By performing data fitting analysis using software, the corrosion current density (I_corr_), corrosion potential (E_corr_), and corrosion rate (Corr. Rate) were determined for each sample and are presented in Table 3. It was observed that the untreated sample exhibited the highest corrosion resistance. The untreated sample displayed an Icorr of 1.44 × 10^−6^ A/cm^2^, an E_corr_ of −0.60 V, and a Corr. Rate of 1.67 × 10^−1^ mm/a. In contrast, the nitrided samples exhibited an increase in corrosion current density by one order of magnitude, a decrease in corrosion potential, and an increase in corrosion rate. The polarization curves of the nitrided samples exhibited noticeable passivation regions. Surprisingly, the Corr. Rate of the LTPN sample was nearly identical to that of the untreated sample. It suggests that the low-temperature nitriding treatment had minimal impact on the corrosion resistance of the samples [56].

The untreated sample exhibited passivation at a corrosion current density of 1.64 × 10^−6^ A/cm^2^, leading to a significant reduction in the ability of the corrosive medium to erode the substrate. However, as the current density reached a value of 1.85 × 10^−6^ A/cm^2^ and the corrosion voltage reached 0.25 V, the initiation of pitting corrosion became observable. The relationship between current density and electrode potential followed an exponential growth pattern, which led to the matrix’s accelerated dissolution and corrosion rate.

The LTPN sample displayed clear passivation at a corrosion current density of 1.44 × 10^−5^ A/cm^2^. The passivation zone widened significantly, the self-corrosion potential increased and the corrosion current density decreased. These observations indicate that the passivation film formed on the nitrided layer was stable, thus enhancing the pitting corrosion resistance. However, as the corrosion voltage reached about 1 V, the initiation of pitting corrosion became observable. The polarization curves of the MTPN and HTPN samples exhibited passivation phenomena with similar widths of the passivation zones. However, the polarization curve of the MTPN sample exhibited periodic activation-passivation fluctuations. At an approximate corrosion potential of 0.25 V, the onset of pitting corrosion occurred, triggering an acceleration in the corrosion process. The potentiodynamic polarization properties of the different samples can be ranked as follows: Untreated < HTPN < MTPN < LTPN.

**Figure 11:**
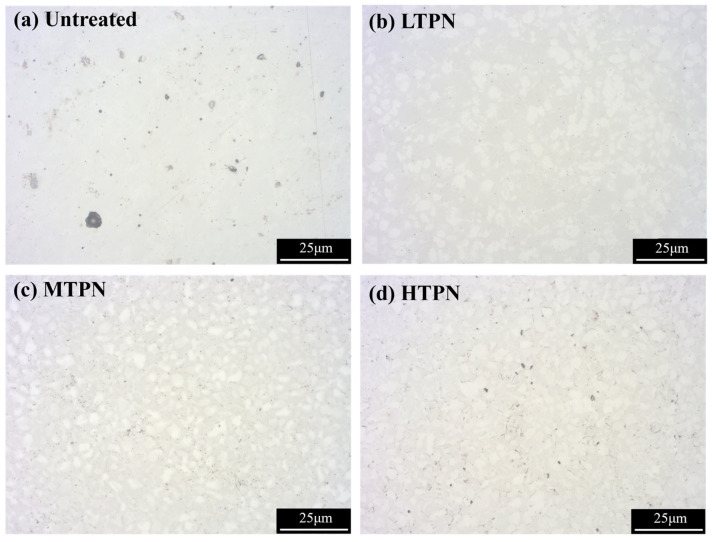
OM micrographs after anodic polarization tests for the untreated and treated samples: (**a**) untreated, (**b**) LTPN, (**c**) MTPN and (**d**) HTPN.

The corroded surfaces of the samples were observed using an optical microscope, as depicted in Figure 11. The surface of the untreated sample appeared relatively flat, with small corrosion pits. The passivation film effectively protected the substrate, preventing further erosion by chloride ions [57]. Small and deep pitting pits were observed on the untreated surface due to the penetration of corrosive media into surface defects and the dissolution of the matrix. This phenomenon is consistent with the polarization curve results shown in Figure 10. The surface of the LTPN sample exhibited corrosion, characterized by numerous punctate pits. However, the LTPN nitrided layer provided adequate protection for the substrate. These pitting pits were minor and did not significantly affect the sample. H. Baba [58] proposed that the oversaturated interstitial nitrogen atoms within the S-phase can enhance the pitting stability of austenitic stainless steel. The nitrided processing exhibited a reliable protective film in the presence of the S-phase. Notably, as the nitriding temperature increased, the pitting pits on the surfaces became deeper. On the surface of the HTPN sample, the pitting pits began to enlarge, indicating the precipitation of CrN under the influence of high temperature, leading to a decrease in corrosion resistance.

**Figure 12:**
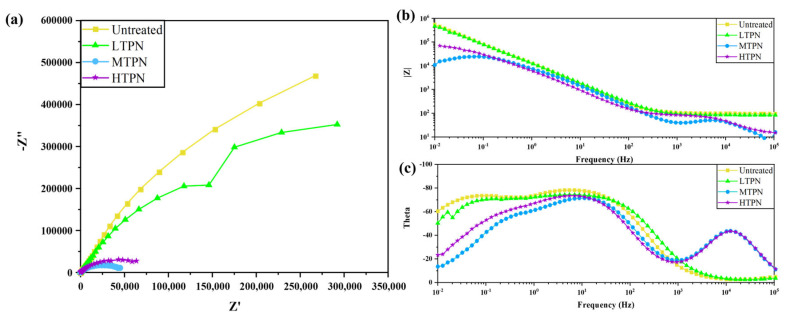
EIS results of the untreated and treated samples in 3.5  wt% NaCl solution: (**a**) Nyquist plot, and (**b**,**c**) Bode plot.

Figure 12 presents the impedance diagrams for the different samples. In Figure 12a, the Nyquist diagram of the sample is depicted, featuring a semicircular capacitance arc attributed to the dispersion effect. Through comparison, it was determined that the untreated sample exhibited the best corrosion resistance in its initial state. Although the LTPN sample displayed a slightly smaller capacitive arc radius than the untreated one, it was still much more significant than those of the MTPN and HTPN samples.

As depicted in Figure 12b, the Bode diagram illustrates the relationship between the log frequency and the impedance modulus. It was observed that all samples in the low-frequency region exhibited the highest modulus. The impedance at low frequencies represented the samples’ polarization and charge transfer resistance. The untreated and LTPN samples showed close similarities in their impedance behavior. Furthermore, the impedance slopes of the untreated and LTPN samples remained relatively consistent within the 10^3^–10^5^ Hz frequency range, demonstrating their frequency-independent characteristics. In contrast, the MTPN and HTPN samples displayed a decrease in their modal values, indicating a downward trend within the frequency range of 10^3^–10^5^ Hz.

In Figure 12c, the Bode phase angle diagram represents the relationship between the log frequency and the phase angle amplitude. For the MTPN and HTPN samples, the phase angle remained around 70°, exhibiting a narrow range. The untreated and LTPN samples demonstrated similar phase angles, approximately 80°, with the broadest range slightly deviating towards the low-frequency region. At the same time, the capacitive response of the passivation film was more substantial over a wide frequency range, exhibiting near-capacitance characteristics. These observations indicate the presence of a highly stable and robust protective and inhibitory passivation film. Additionally, the Nyquist diagram demonstrates that as the radius of the bulk react arc increased, the frequency range of the highest phase angle shown in the Bode diagram widened, signifying enhanced corrosion resistance. The order of corrosion resistance among the samples is as follows: MTPN < HTPN < LTPN < Untreated.

**Figure 13:**
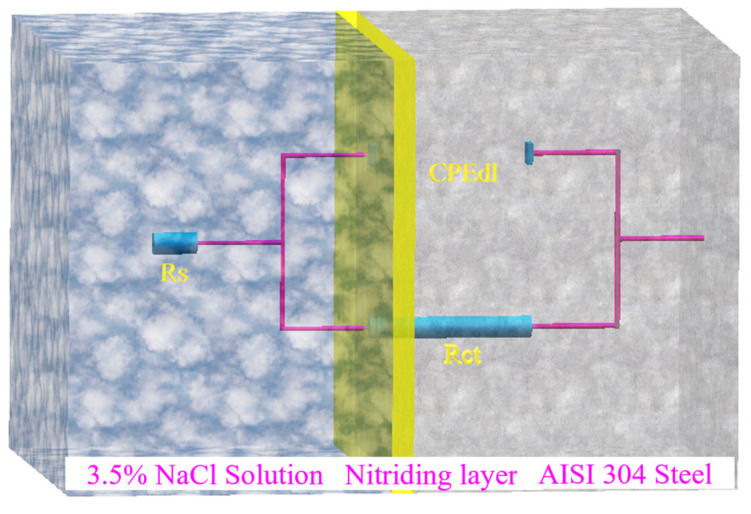
Equivalent circuit of the nitrided samples in 3.5% NaCl solution.

The impedance Nyquist diagram was analyzed to establish and fit the equivalent circuit, as depicted in Figure 13. The results of the fitting parameters for the equivalent circuit are provided in Table 4. In the equivalent circuit of all samples, the electrolyte solution resistance (R_s_) represents the resistance of the electrolyte solution. In contrast, the charge transfer resistance (R_ct_) and constant phase element for the double-layer capacitance (CPE_dl_) characterize the charge transfer resistance and the electric double-layer capacitance at the interface between the electrolyte solution and the material matrix.

Notably, the values of CPE_dl_ for the untreated and LTPN samples were very similar, measuring 1.73 × 10^−5^ and 1.76 × 10^−5^ F·cm^−2^, respectively. However, the R_ct_ value of the untreated sample (1.61 × 10^6^ Ω·cm^2^) was approximately 1.5 times higher than that of the LTPN sample (0.98 × 10^6^ Ω·cm^2^). Furthermore, the MTPN and HTPN samples exhibited lower CPE_dl_ values of 4.87 × 10^−5^ and 5.37 × 10^−5^ F·cm^−2^, respectively, along with lower R_ct_ values of 4.67 × 10^4^ and 7.41 × 10^4^ Ω·cm^2^. These values were significantly lower in comparison to the untreated and LTPN samples.

**Figure 14:**
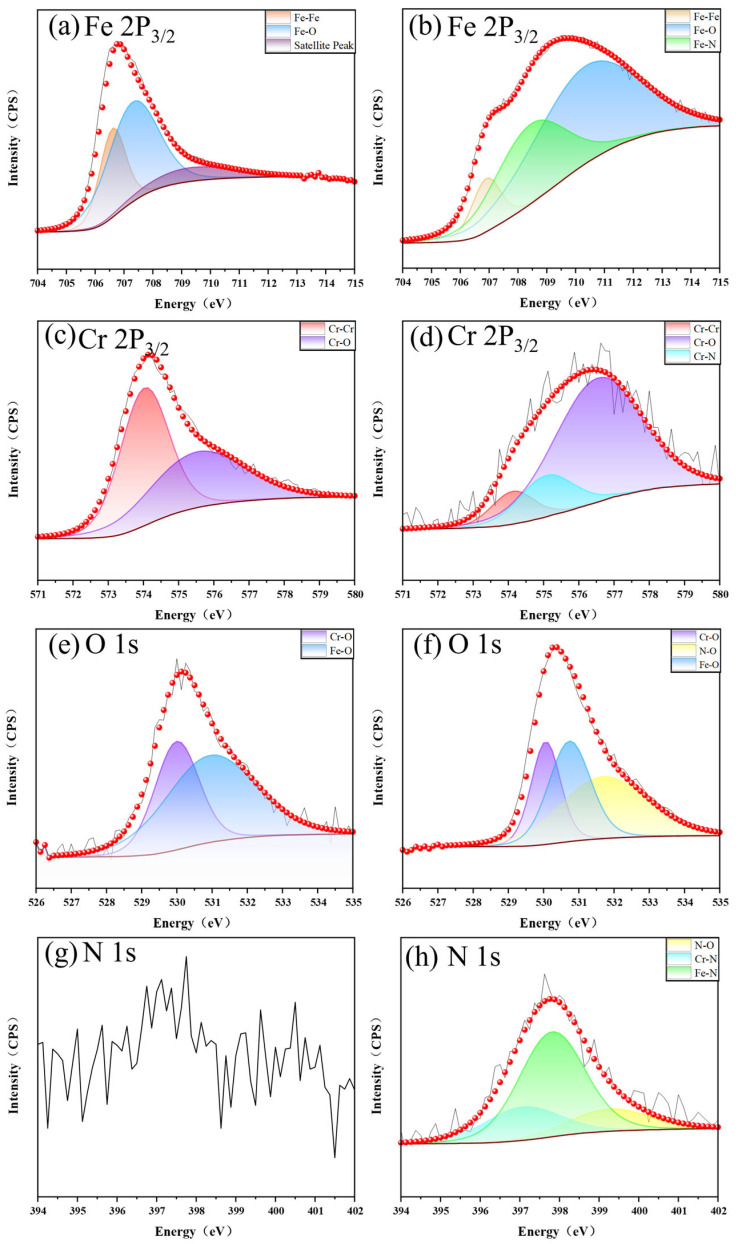
The XPS energy spectrum of the corrosion surface of different samples: (**a**,**c**,**e**,**g**) are the XPS spectrum of the untreated sample; (**b**,**d**,**f**,**h**) are the XPS spectrum of the LTPN sample. The red dots are the fitted curves and the black lines are the original curves.

The untreated and LTPN samples underwent XPS analysis following the electrochemical corrosion test, focusing on the elements Fe, Cr, O, and N. Figure 14 presents the XPS fitting spectra of the corrosion surfaces for both the untreated and LTPN samples. The graphical representations in Figure 14a,b illustrate the outcomes of the fitting process for the Fe 2p 3/2 peak detected on both the untreated and LTPN samples. The presence of metallic iron (706.6 eV) and ferrite (707.3 eV) was observed in Figure 14a. The peak at 709.3 eV corresponds to the satellite peak of trivalent iron compounds [59]. In addition, the presence of metallic iron (706.9 eV) and iron oxide (710.3 eV) was confirmed through the analysis depicted in Figure 14b.

Furthermore, iron nitrides were observed at 708.4 eV, reflecting the effect of the nitriding treatment on the LTPN sample. The penetration of nitrogen into the matrix facilitated the generation of iron nitrides with iron. The fitting results for the Cr 2p 3/2 peaks detected on the untreated and LTPN samples are depicted in Figure 14c,d, respectively. Both metallic chromium (574.0 eV) and chromium oxides (575.5 eV) were identified in Figure 14c. The presence of metal chromium (574.1 eV) and chromium oxide (576.5 eV) was also confirmed. The oxide form of chromium in this position is considered to be Cr_2_O_3_ [60]. Additionally, a small amount of chromium nitrides (575.1 eV) were found in Figure 14d [60,61,62]. The fitting results for the O 1s peak on the surfaces of the untreated and LTPN samples are presented in Figure 14e,f. Iron oxides (530.9 eV and 530.7 eV) and chromium oxides (530.0 eV) were observed in both spectra. Evidence of nitrogen oxides (531.7 eV) was also detected in the O 1s spectra of the LTPN sample (Figure 14f). Figure 14g,h display the fitting results for the N 1s peak on the surfaces of the untreated and LTPN samples. Since the untreated sample (Figure 14g) was not nitrided, the presence of N was not found. However, in Figure 14h, nitrogen oxides (399.3 eV), iron nitrides (397.8 eV), and chromium nitrides (397.1 eV) were detected [63]. It can be observed that the content of chromium nitrogen compounds is relatively low, which corresponds to the peak of chromium nitrogen compounds in Figure 14d. The dominant peaks in both samples are associated with oxides, resulting from the corrosion of the sample surfaces and the generation of a significant number of oxygen and matrix elements.

**Figure 15:**
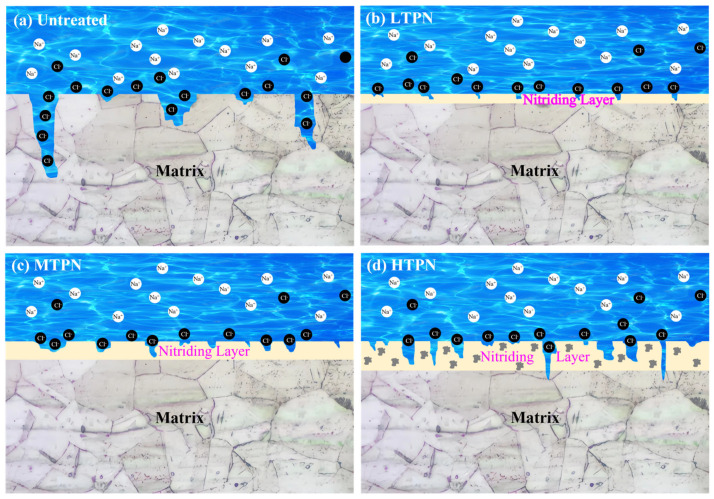
Schematic illustrations of corrosion behavior of different samples in 3.5% NaCl solution.

A schematic diagram illustrating their corrosion behavior was developed based on the polarization curve (Figure 10) and the corrosion morphology diagram (Figure 11), as presented in Figure 15. When exposed to the air, the untreated sample formed a thin passivation film, protecting the substrate [64]. By analyzing the polarization and the impedance, it can be seen that the initial state of the untreated surface was the best, which was manifested by its high corrosion potential, low corrosion current density, and good impedance performance. This favorable initial state was due to the presence of the passivation film. However, during the electrochemical corrosion test, chloride ions (Cl^−^) could infiltrate the defects and disrupt the passivation film, forming acidic corrosive substances involving Cl^−^ and O^2−^. These substances corroded and dissolved the matrix, causing pits on the untreated surface (Figure 11). This passivation film underwent dissolution in the presence of aggressive ions, thereby exposing the underlying material and initiating a subsequent passivation-corrosion cycle. Due to its lower potential in the electrolyte solution, the exposed material served as the anode during the dissolution of the passivation film, and the undissolved passivation film acted as the cathode [65]. This formation of micro-galvanic corrosion accelerated the overall corrosion process. Under the influence of an acidic environment, the nitrogen atoms within the S-phase layer were released, effectively neutralizing the acidic ions. This process prevented excessive acidification of the microenvironment within the pitting corrosion, thereby creating favorable conditions for the self-repair of the passivation film.

The LTPN sample demonstrated excellent corrosion resistance in a 3.5% NaCl solution. The presence of the S-phase made the surface rich in N. Through the reaction [N] + 4H^+^ + 3e^−^ → NH_4_^+^, nitrogen forms ammonium ions (NH_4_^+^), facilitating surface passivation and consuming chloride ions [66,67]. XPS analysis revealed the formation of Cr_2_O_3_ on the surface of the nitrided sample after corrosion. Dense Cr_2_O_3_ acts as a beneficial substance, forming a protective barrier [68,69].

However, after the nitriding treatment, the surface of the sample underwent the formation of the S-phase, which predominantly comprised nitrides and was extensively coated with a substantial accumulation of sputtered material. The surface became intricate, making it challenging for some aspects of the air to replace its composition and form a passivation film. Moreover, the nitriding treatment led to increased surface roughness and defects, contributing to the unsatisfactory performance of the nitrided samples in the initial stage of the electrochemical corrosion test. This phenomenon was confirmed by the nitrided samples’ polarization curves (Figure 10) and impedance spectra (Figure 12). Initially, the nitrided samples exhibited a low corrosion potential, high corrosion current density, and poor impedance performance. However, as the electrochemical corrosion test progressed, the nitrided samples demonstrated a passivation phenomenon with a wide passivation zone. Furthermore, in the latter stage of the electrochemical corrosion test, no accelerated dissolution corrosion was observed, indicating the quick formation of a passivation film to protect the substrate when chloride ions penetrated the S-phase of the nitrided samples.

As the nitrided temperature increased, the S-phase layer gradually thickened, and CrN began to aggregate. More pits appeared on the surface of the MTPN sample, yet the corrosive medium still did not penetrate the protective S-phase. The HTPN sample developed a thicker nitrided layer. However, a significant amount of CrN precipitated due to the excessive temperature, leading to reduced corrosion resistance. Although pitting persisted on the surface, the resulting pits exhibited smaller dimensions and shallower depths for the nitrided samples [65].

The presence of the Cr_2_O_3_ substance in the XPS diagram (Figure 14) provides evidence for the formation of the passivation film. The existence of the S-phase contributed to protecting the nitrided samples during the mid and late stages of the electrochemical corrosion test. OM examination revealed no severe corrosion on the nitrided surface. However, CrN accumulation occurred with the increase in nitriding temperature, gradually reducing the corrosion resistance. In the later polarization stage, the corrosion current density gradually increased, which was concomitant with an escalation in quantity of pits. CrN precipitation of the HTPN sample decreased the concentration of free Cr in the S-phase layer and insufficient Cr element for passivation film formation. The HTPN sample displayed the weakest corrosion resistance among the nitrided samples.

## 4. Conclusions

(1) The hollow cathode-assisted plasma nitriding method can improve the plasma density through the hollow cathode geometry, obtain more active N atoms quickly, and improve the efficiency of plasma nitriding. We believe 450 °C is the best process temperature for hollow cathode plasma nitriding. It can quickly obtain the S-phase of good corrosion and friction resistance. Compared with conventional plasma nitriding technology, the efficiency is improved by about five times;
(2) The LTPN sample had the best mechanical and frictional properties. The hardness of the LTPN sample was increased by nearly three times, the H^3^/E^*2^ value was increased by nearly 30 times, the COF was reduced by 21.5%, and the wear rate was reduced by 99.8%;
(3) The corrosion resistance of nitriding samples was poor in the early stage of electrochemical corrosion. With the corrosion process, a passivation film was formed on the surface of the nitriding sample, which prevented the intrusion of the corrosive medium. The corrosion resistance of nitrided samples was better than that of untreated samples in the later stage. With the increase in nitriding temperature, the phenomenon of “Cr poverty” began to occur, and the corrosion resistance of the nitrided samples decreased.

## Figures and Tables

**Table 1:** Chemical compositions (wt.%) of AISI 304 steel.

C	Cr	Ni	Mn	Si	S	P	Al	Fe
0.06	18	8.5	1.8	0.36	0.005	0.018	0.015	balance

**Table 2:** Wear volume and specific wear rate of the untreated and nitrided samples.

Sample	Wear Volume (mm^3^)	Wear Rate (mm^3^/Nm)
Untreated	2.30 × 10^2^	1.92 × 10^−3^
LTPN	4.30 × 10^−1^	3.59 × 10^−6^
MTPN	9.16 × 10^−1^	7.63 × 10^−6^
HTPN	9.34 × 10^−1^	7.78 × 10^−6^

**Table 3:** Electrochemical parameters from the polarization tests for the untreated and treated samples.

Sample	I_corr_ (A/cm^2^)	E_corr_ (V)	Corr. Rate (mm/a)
Untreated LTPN	1.44 × 10^−6^ 1.47 × 10^−5^	−0.60 −1.03	1.67 × 10^−1^ 1.70 × 10^−1^
MTPN HTPN	6.51 × 10^−5^ 2.18 × 10^−5^	−0.93 −0.98	7.54 × 10^−1^ 2.52 × 10^−1^

**Table 4:** Electrochemical parameters of the untreated and treated samples.

Sample	R_s_ (Ω·cm^2^)	R_ct_ (Ω·cm^2^)	CPE_dl_ (F·cm^−2^)
Untreated LTPN	95.63 81.15	1.61 × 10^6^ 9.80 × 10^5^	1.73 × 10^−5^ 1.76 × 10^−5^
MTPN HTPN	48.72 50.55	4.67 × 10^4^ 7.41 × 10^4^	4.87 × 10^−5^ 5.37 × 10^−5^

## Data Availability

The data presented in this study are available on request from the corresponding author.
